# Applicability of Vfrac in men: a qualitative study of an osteoporotic vertebral fracture screening tool for use in older people with back pain

**DOI:** 10.1007/s11657-024-01470-8

**Published:** 2024-11-19

**Authors:** Karen L. Barker, Francine Toye, Sarah Drew, Tanzeela Y. Khalid, Emma M. Clark

**Affiliations:** 1 Nuffield Department of Orthopaedics, Rheumatology and Musculoskeletal Sciences (NDORMS), University of Oxford, Botnar Research Centre, Oxford, OX3 7LD UK; 2 Physiotherapy Research Unit, Oxford University Hospitals NHS Foundation Trust, Oxford, UK; 3 Musculoskeletal Research Unit, Translational Health Sciences, Learning and Research Building, Southmead Hospital, Bristol Medical School, University of Bristol, Bristol, BS10 5NB UK

**Keywords:** Vertebral fractures, Back pain, Osteoporosis, Vfrac

## Abstract

**Summary:**

The Vfrac clinical screening tool was developed to help primary care healthcare practitioners decide if an older woman with back pain is at high risk of a vertebral fragility fracture (VFF) and requires a spinal radiograph to confirm diagnosis. The Vfrac tool developmental work was carried out in women because of the higher background prevalence of VFF. We now wish to assess Vfrac in men.

**Purpose:**

To understand and characterise pain symptoms of men with VFF, to evaluate the wording of the Vfrac tool from men’s perspective, and to establish if a gender-specific version of the Vfrac tool was needed.

**Methods:**

Individual interviews were conducted with 15 men using an interview topic guide based on the original Vfrac topic guide with the addition of a ‘think aloud’ section to discuss the wording of the current questions within the Vfrac tool. Thematic analysis was conducted by two researchers.

**Results:**

Seven themes highlight that physical measurements can be potentially upsetting for those being measured (‘Weighed, measured and found wanting’), that closed questions cannot capture the complexity of experience (there is no room on the paper; pain is dynamic, not static; walking can make it better or worse; well, it depends on which chair), and that gendered roles are varied and dynamic (I try to do my share of domestic work; no more do-it-yourself).

**Conclusions:**

This research has allowed the male perspective of osteoporosis to be heard and importantly identified that the Vfrac tool had no gender-specific barriers.

**Supplementary Information:**

The online version contains supplementary material available at 10.1007/s11657-024-01470-8.

## Background

### Introduction

Osteoporosis and associated fragility fractures are one of the most common musculoskeletal conditions in older people, with vertebral fragility fractures (VFFs) of particular importance, as they identify people at high risk of future fracture. Within 5 years of their occurrence, one in four people will have a further vertebral fracture, and one in ten will have a limb fracture (including hip fracture) [1]. VVFs lead to morbidity and disability: more than a third of patients with a VVF have difficulties with activities of daily living for the rest of their life [2, 3].

Identifying VVFs provides the opportunity to intervene with bone protection therapies that can reduce the risk of further fractures by 30–65% [4–6], but an estimated two-thirds of VFFs are undiagnosed [7]. People with existing VFFs are missed for a variety of reasons. A major reason is lack of understanding of which clinical features should be used as triggers for referral for diagnostic spinal radiographs in patients with possible VFFs [8]. An approach is needed to address the lack of awareness of clinical triggers for referral by GPs and other first contact health care practitioners for diagnostic spinal radiographs in the first place. Because of the high prevalence of all-cause back pain, GPs are generally dissuaded from referring patients for radiographs [9], and, without accurate clinical indicators for VVFs, most patients with these fractures are likely to remain undiagnosed until presenting with late clinical sequelae.

There has been progress in understanding the characteristics of VFFs to assist in the development of screening tools. For instance, Clark et al. (2016) showed that clinical indicators such as presence of back pain in combination with other features such as history of fracture can assist in identification of older women at risk of prevalent osteoporotic vertebral fracture [10]. It has also been shown that the site of back pain can indicate the likelihood of VFF [11]. Although these indicators have the potential to enhance identification of women with VFF, it remains uncertain whether pain characteristics might provide useful indications of pain related to VFF rather than other causes.

To address the problem of under diagnosis, Clark et al. developed the Vfrac clinical decision tool using the Medical Research Council (MRC) framework for development and evaluation of complex interventions [12, 13]. The intention of Vfrac was to help healthcare practitioners in primary care decide if an older woman with back pain is at high risk of a VVF and therefore requires a spinal radiograph to confirm the diagnosis. It is intended to be used in either a face-to-face or telephone consultation between a health care practitioner and older person presenting with back pain. It consists of 15 simple components based on self-reported data and a physical examination. It takes less than 5 min to perform and produce a binary output of ‘Low risk—spinal X-ray is not recommended’ or ‘High risk—spinal X-ray is recommended as may have a vertebral fracture’.

However, all of the Vfrac tool developmental work has been carried out in women because of the higher background prevalence of VVF. Similarly, the work characterising pain description with VFF has also been with women [14]. Whilst, one in five men will sustain one or more osteoporotic fractures in their lifetime [9], most of the research reporting the experience of people with VFF has excluded the perspective of men. We now wish to assess Vfrac in men. There is clear evidence that moderate and severe VVFs in men predict future hip fractures in a similar way to women [15]. There is support for the assumption that the underlying biology of osteoporosis and VVFs is similar in men and women [16, 17]. Pharmaceutical studies show that bone protection therapies work equally well in men and women in protection against future VVFs [18, 19]. However, there remain questions around men’s characterisation of back pain symptoms in the context of VVF.

Therefore, the aims of this study are to understand characterise experiences of pain and other physical symptoms of VFF in men and to understand men’s views of the wording of the questions in the current Vfrac tool.

## Methods

### Participants

Men who were over the age of 65 and had a confirmed diagnosis of a vertebral fragility fracture by spinal radiograph or MRI from North Bristol NHS Trust or University Hospitals Bristol and Weston NHS Foundation Trust were invited to participate. Second, men with a confirmed diagnosis of VFF who had consented to be included in a database of people willing to be involved in research were contacted by researchers from University of Oxford.

### Approaching and recruitment

Ethical approval was obtained from London- West London & GTAC Research Ethics Committee (reference: 22/PR/0088). Patients were sent an invitation pack which consisted of an invitation letter, information booklet, two copies of the consent form, and a freepost envelope to return to the study team. They were asked to contact the study team if they were interested in taking part. They were given the option to have an interview in person, on the telephone, or via a secure video platform (on Microsoft Teams). Potential participants were then contacted, any questions they had were answered, and consent to participate was completed.

### Interviews

**Fig. 1:**
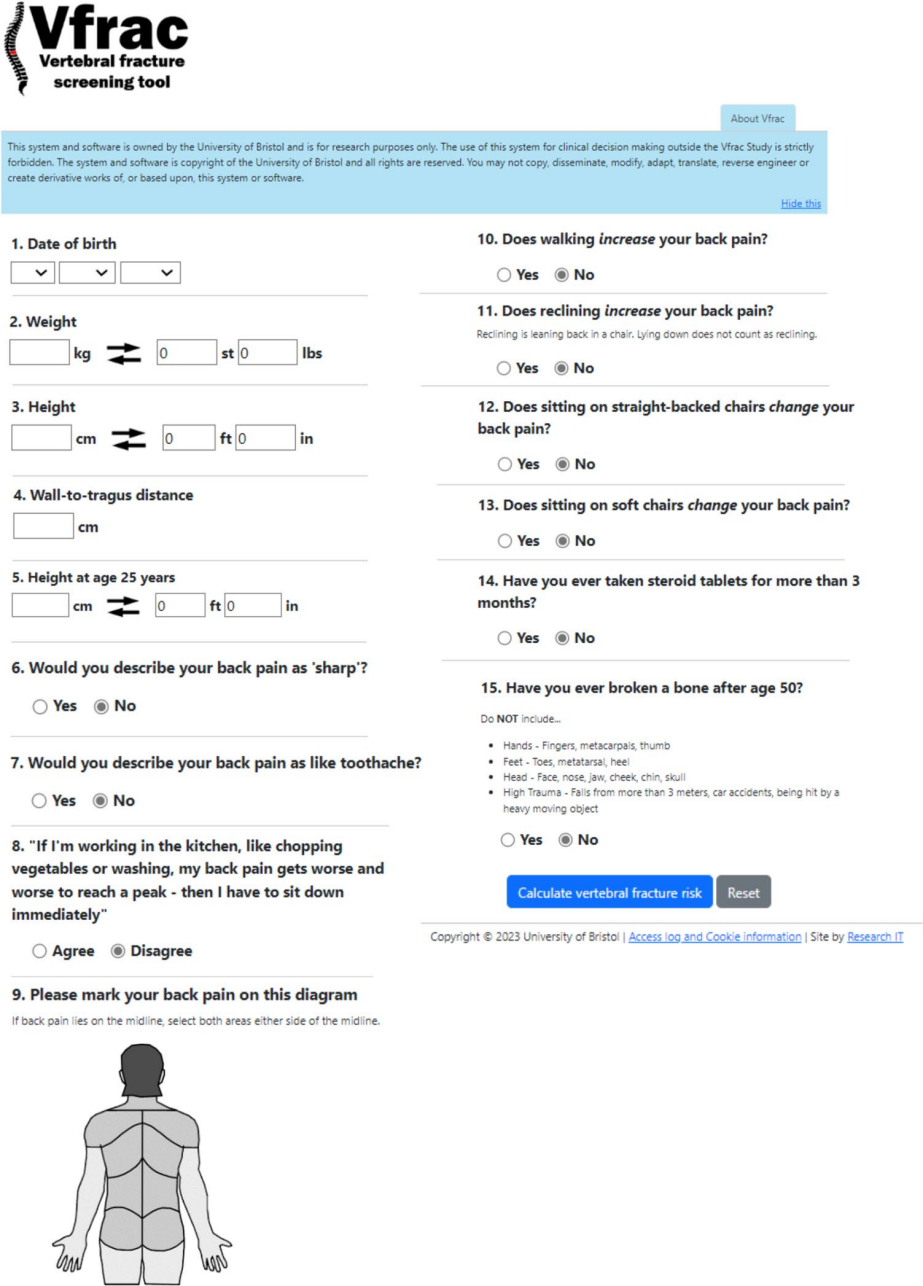
Vfrac tool

The study team developed an interview topic guided aiming to focus on men’s experiences of osteoporosis based on the original Vfrac topic guide (Appendix 1) with the addition of a ‘think aloud’ section to discuss the wording of the current questions within the Vfrac tool (Fig. 1). Think aloud interviews are a type of qualitative interview where participants engage with a stimulus material/intervention and say out loud out loud thoughts that come to mind as they work through it. This allows capture of detailed data about how users react to the intervention and how they interact with it [20].

A printed version of the Vfrac tool was used as a stimulus material to some of the questions (Fig. 1).

Interviews were one to one and used the topic guide flexibly to explore the impact of osteoporosis on men’s lives; friends and relationships, emotions and feelings, recreation and fun, work and household roles, and any other areas identified by the men. Interviews were conducted by FT and KB, both female post-doctoral researchers one with a background in physiotherapy and one anthropology. Interviews were recorded, transcribed verbatim, anonymised, and checked for accuracy. They were then imported into NVivo qualitative analysis software to assist organisation of coding and thematic analysis.

### Data analyses

Data were analysed using a thematic approach to identify themes in the responses and summarise reflections [21]. Thematic analysis was conducted with transcribed data initially coded by one researcher (FT) and independently by another member of the research team (KB). To ensure rigour, independent coding was discussed and further refined. Transcript data were broken down into discrete units, coding each meaning unit to remain close to the data.

Findings were compared to previous work conducted within the team to characterise women’s experiences of VVFs [22].

## Results

We interviewed 15 men aged 63–94 years (median 82).

Interviews varied in length from 56 to 92 min. Participants were given the option to have an interview in person, on the telephone, or on Microsoft Teams. Only one participant requested a phone interview. All other interviews took place in participant’s homes.

We present themes that help us to understand the complexity and ambiguity involved in completing survey questions as part of a diagnostic interview.

### Weighed measured and found wanting

This theme describes the impact on participants of being weighed and measured as part of an assessment (questions 2–5). For some, the moment of being weighed and measured could be shocking, upsetting, or concerning, although this did not necessarily align with a tendency to joke or make light of the situation.

I didn’t want to sort of milk it, but I mean I was shocked; not today doing it because I’ve known this for a while, I’m shocked to see how much I’d gone down in height, in fact I think it was when she measured me, I thought she’d done it wrong … so that was a bit of a shock.

One thing that’s very striking is, [the VFRAC] says, ‘How tall are you today?’ I'm 5’ 7½”, my wife did my height for me this morning, and that is a full four inches shorter than I was a few years ago. In the past I've always measured myself as 5’ 11½”, so I've lost a lot of height.

When asked to measure the distance between the ‘tragus’ [on the ear] and the wall (question 4), none were able to do this without assistance. Some were concerned that this might indicate that there was something seriously wrong with them (‘it says in there you can do it on your own!’). This tangible ‘proof’ of spinal deformity left one participant feeling humiliated.

I [just] couldn’t do it! It points it out to me. I couldn’t understand why – I thought it must be easy – just stand against the wall. My head will not go back to the wall – not unless I do ‘That.’ … cannot do it. So, I’m assuming that the top of my spine has got a curvature on it, so my head’s further away from the wall. ….. Well, it says in there you can do it on your own! It’s not possible!

I remind myself of the two people who used to be on that traffic sign you know beware old people crossing. [laugh] Cos I know that this is probably the way I’m going to be because they can’t straighten me up anymore.

### There is no room on the paper

This theme explores the ambiguity of answering questions; ‘not everyone would understand the questions’. Although some felt that completing the tool on their own would save time, others wanted to talk through the answers (‘what did you mean by that?’). Some felt that questions were leading or even misleading, and that they did not allow you give a ‘true’ and complete answer.

there’s an ambiguity in the question quite often … I like to be able to challenge generally, ‘what did you mean by that?’ So that I can try to give you a precise answer … the people writing the questions have got some picture in mind, but they should be trying to look at the question they’ve written down and then think about what the average people might read into this … sometimes prejudices can be built into the question.

Some explained that if a question was not clear, they would try to understand it within the context of the questions around it.

Often if I’m unsure on a questionnaire I’ll go on to the next one and the next one because it will tell me the answer to the one previous, well or it’ll lead me the way to the one previous. … because often you find that questions follow on from other questions.

However, although sometimes frustrated by not having the chance to give a full and precise answer, participants understood that questionnaires could only be a proxy for full accounts.

Talking to you [about the questions and answers] there’s a kind of feedback whereas if you’re filling a form in you’ve got no-one you can refer to … I think doing it the way you’ve done it this afternoon has been quite good … there wouldn’t be room on the paper probably to write it all down.

### Pain is dynamic, not static

This theme is underpinned by the ambiguity of pain descriptors, ‘sharp’ and ‘toothache’, and the challenge of finding the right words to describe pain: ‘I can’t think of the right words’; ‘It’s difficult to give a straight answer’. Participants described pain as variable, fluctuating, and contextual: pain could be both sharp *and/or* dull, nagging *and/or* fleeting, present *and/or* absent, and localised and/or radiating. There was a sense that the words ‘sharp’ and ‘toothache’ could not fully encompass pain experiences.

[Q: is your pain sharp or like a toothache?] Well, neither of those things were true for me, and I tried to give you a description of what the pain is like. And that description doesn't come anywhere near what it says in this form.

Toothache can come in two ways, can’t it? Because it can come out as a searing pain if you have a hot drink on a nerve or something but also toothache can just be a sort of throbbing pain which is what mine is more like now … It’s that dull aching.

The essential quality of toothache was its ‘wearing’, ‘drawn [out]’, or ‘relentless’ quality which distinguished it from fleeting or intermittent pain.

It’s wearing, you know, toothache is wearing. And you can forget about it and then you come back to it … I felt the allusion to toothache as a way to think about it was quite useful actually.

I don’t know how you’d describe that pain, really? It’s a dull sort of ache that’s there all the time. There’s no escape from it … it’s a nagging pain that’s there all the time and you can’t get away from it. So yes, it is similar [to toothache], isn’t it?

Some found it difficult to qualify pain because pain varied depending on context, time, and frame of mind: ‘Some days I can skip like a lamb [and other days…]’.

In some ways it’s a bit more complicated because it affects me in different ways at different times in any one day…. It’s sort of … I never get a static pain you know one that just goes on and on and never changes, it’s always quite dynamic … sometimes I can skip around like a lamb; other times I’m sort of shuffling around like a penguin … if you catch me today, I’m okay apart from that slight niggle, you get me at another time, and I might have a pain down the back of my leg.

Some considered that you could acclimatise to pain; others found it difficult to unpick osteoporotic pain from the bricolage of other existing co-morbidities, such as osteoarthritis.

When it [first] happened it was agony, I mean it really was intense the pain … after probably eight weeks … it was still there, but it was perfectly manageable … now . [its] just a big, dull, thudding ache and I suppose you just end up living with it and becoming used to it.

Well, as I’m sitting here, I’ve got a dull pain, ache. But as I described, I can go on the stairs and suddenly get a sharp pain. Whether that is actually to do with osteoporosis or whether that’s to do with arthritis, I don’t know.

For some, the body diagram did not cover all areas affected by pain (question 9), and this made it difficult to map onto real body experiences. For example, the body diagram did not differentiate localised, central, radiating, anterior, or side pain. Some found it difficult to decipher the front from the back, or left from right view on the body diagram.

Is that the view from the back? I thought it was the front because the hands look like they’re facing forward, don’t they? Well, it does say ‘back’ at the top … but actually it says ‘back’ where you might have written the word ‘top’! [laughs]… All the parts of the body are not there, so do people get osteoporosis in all parts of the body?

so that’s the left side and right side? … [I suppose you can] see from the feet…

### I try *to do* my share of domestic work

This theme explores gendered roles in the household. Participants related to the question: ‘If I’m working in the kitchen, like chopping vegetables or washing, my back pain gets worse and worse to reach a peak—then I have to sit down immediately’ (question 8). Several participants enjoyed cooking and took a share of kitchen and housework. In some households, men took charge of cooking, and in other households, women assumed the bulk of domestic work. Participants who had lost their wives, now cooked, and cleaned for themselves.

I still do a lot of cooking. I tend to cook the evening meal virtually every day, and my wife does more long-term cooking like cooking meals to go in the freezer for future use, and that kind of thing. But I do the regular evening meal, and it hasn’t affected that at all. I also do a lot of washing-up and drying up, and so on.

I do some cooking, yeah, not all of it. I keep ready meals in the freezer … laundry, I do most of my own. I mean, my wife and I, we always shared all the domestic duties all the way through, and she helped out in other things as well, that’s just the way it was. So, we both just sort of got stuck in and did it.

Participants were aware of gender-stereotyped domestic roles. Some talked about these with humour, acknowledging that their wife might put them ‘to shame’ because they did more than their fair share of domestic work.

I do lots of cooking. [*facetious*: both laugh] It’s the standing peeling the veg and that, that’s what I can't do [*again facetious*]. [laugh] … And washing the pans and that sort of thing. I don’t like cooking, no and my wife is such a good cook that. She puts me to shame. But I do lots of other things around the house … I do all the hoovering. … my wife loves cooking, and I love eating.

I mean I’m from an age when women did most of the work around the house and unfortunately, I’ve relaxed into that to an extent cos my wife is very capable.

Some described less-stereotypical domestic roles, grounded in formative experiences. For example, if mum had to earn money whilst dad was on active service during wartime, children took on caring and household duties.

I’ve always been tidy … It’s just the way I’ve always been. I was brought up, I was the second oldest in a family of eight… when mum had to go to work to bring in a few extra pounds a week – we used to get the younger ones up and ready for school while mum was out doing office cleaning and things like that … [collecting sticks] was a job that I had to do for my two nans to keep wood for their fire.

However, domestic work could be painful, and having to lean on surfaces to take the weight off the spine meant that kitchen work was not only painful but also slow and tiring.

Jobs take you twice as long because you’re leaning with one hand to support yourself … you’re not going to the cupboard to get two cups you’re going to be the cupboard and getting one cup out, and then another cup out … cos you need the support of the other arm to keep you upright.

### No more do-it-yourself

This theme describes the erosion of practical or ‘manual’ jobs which some would have ‘naturally done’ themselves. Even those who did not ‘cook and chop’ (question 8) were able to align this with their own experience of other household tasks such as painting, decorating, and household repairs.

Your survey asked about if you were standing there chopping vegetables … I’m a keen gardener, potting up seedlings … wheeling a wheelbarrow and digging stuff, that sort of thing, I seem to cope with reasonably well but certain positions … I could stand there, and it doesn’t take long for me to start hurting if I stand.

I do woodturning in my shed and that involves standing and the same thing applies. I can only do it for so long, and then I have to stop, have a break …being static is the worst thing for me. If I'm moving around or keeping active, then I'm okay but if I'm static for any period of time, then my back does ache.

Relinquishing activities and tasks could be a heavy loss. Again, there was a tendency to talk lightly, even about losses that were deeply felt.

I did a lot of DIY [do-it-yourself] work … We’ve still got a room that needs decorating… I enjoy the painting and the decorating, all of it, and I just can’t do it anymore … I think ‘God I wish I’d have done this; I wish I’d have managed to get this done’ … I would be naturally doing that myself yeah … I used to clean windows. [laugh] We now have a window cleaner.

I’m not as good on the practical things … I can’t lift the weights I used to, nor can I do jobs around the house so much as I used to, house maintenance and that kind of thing … If we needed a new loo seat I would go to a shop and buy one, and then assemble it myself. But I can’t do that so easily now because you have to be able to bend down and that kind of thing. I can’t sit on the floor or kneel.

### Walking can make it better or worse

This theme describes the variable impact of walking on pain (question 10). Speed, distance, surface, and amount of time into a walk had a qualitative impact. Some used a walking aid (such as a stick or a walking pole) to take weight off the spine. Other health conditions also impacted on capacity to walk.

I mean walking distances … by the time I’ve got to the top [of the road] I’m sort of aching but just ordinary walking around the house, work, whatever, that doesn’t bother me at all, but if I walk for any sort of length of time, any distance, and if there’s an incline and that sort of thing.

If I walk quite slowly round somewhere ... I can go to the museum and walk around there, and nothing happens, it’s fine. But going out walking the dogs – the dogs will want to walk quite fast, and that really brings it on. I mean … by the time I get to the traffic lights, it’s starting to hurt already … it’s agonising.

For some, walking improved pain because it was a dynamic activity: ‘It seems to release it a bit’.

I start walking a few steps and that would be pain there. Carry on and it gets better … I mean there’s a distinct progression so initially the pains and difficulty bouncing and moving and going through to perhaps almost normal and then it can get painful because you’re using those muscles too much because they haven’t been exercised enough.

For some, walking had been a valued part of life, entangled with socialising and fun and there was a strong incentive to keep walking. This also sets the context for answering this question (number 10).

My wife I are great walkers, we love long walks, but I can’t do long walks anymore, I can just do short ones. So that has made a difference there … We have relatives and they’re great walkers too, I’m unable to go with them you see … The walks are very important because we’ve always loved doing walks, we go regularly for holidays… in the mountains. We still go there but I can’t go on the long walks anymore.

### Well, it all depends on which chair

This describes the ambiguity of questions related to resting position. ‘Reclining’ was understood in different ways (‘you mean lying down?’; ‘I assumed that reclining was going flat’; ‘laying out on the sofa’; ‘vaguely horizontal but not quite’).

Reclining, well it depends … if I was reclining on the floor, the further I attempt to get my back down onto the floor the more painful it would be, but [in this chair] the back gets supported … [but] I’ve got a recliner [chair] and that doesn’t increase the pain; in fact, it eases it to a certain extent.

The impact of reclining on pain hinged on contextual factors such as how far you were leaning back, the quality of the surface; how long you were in that position, or what you were doing. The concept of ‘straight-backed chair’ and ‘soft chair’ also needed clarification.

I don’t like them straight back [chairs] I like a bit of a tilt in them … this has got enough lean back on it … I can support myself on there … you can’t sit on soft chairs … these low sofas and everything nowadays. It’s too soft … it’s uncomfortable.

if you said to me sit here for three or four hours, you know, as you say, it might be in a restaurant or something, then that could cause an issue.

For some, ‘upright’ or ‘soft’ was conflated with ‘hard’ or ‘reclined’. For others, soft or hard could cause either comfort or discomfort (‘it depends’). For example, a straight back could be seen as ‘hard’ and uncomfortable *or* supportive and comfortable. The qualities of *softness/hardness and upright*/reclined were described as dualities on a spectrum: as such, they required clarification. For example, ‘it needs to be firm. But not hard’ where ‘firm’ aligned with comfort and support, and ‘hard’ aligned with discomfort.

I go to church on Sunday, and chairs in the church although they’re padded … I do find myself being a bit uncomfortable in my back, sitting on these particular chairs. They're not hard chairs, but they’re not soft.

[Laughs] Well, some [hard] chairs bend back, don’t they. So, I rather took it to [straight-backed] to mean upright … a hard upright thing? … Well, having a bent back, a hard chair back, very uncomfortable. I’m using a chair with a sort of slatted back, and I’ve tucked a sort of pad between the slats at the back, and that’s been quite a blessing to have a soft place to rest my back.

The challenge of providing a clear answer was exacerbated by the question ‘does it *change* your back pain’, which had no directional qualification (increase or decrease), could leave participants second guessing the meaning of the question.

## Discussion

Our main findings from this study were that the Vfrac screening tool had relevance to men with VFF and that the existing tool, designed based on the experiences of women, was applicable to both. The respondents also raised interesting points about the utility if completed by virtual consultations with more independent answering of questions than the original testing in a face-to-face consultation with a health care provider.

Whilst we did not find any gender-specific problems and so do not need to consider developing a separate men’s Vfrac tool, we did gain further insights into how participants understood and interpreted the questions. It is recognised that men are under-represented in osteoporosis research and most treatment strategies are not gender specific [23]. However, in our study, the men interviewed felt that interpreting the questions in the Vfrac screening tool resulted in differences that came from their individual frame of reference and the way they constructed the sentence as they read it, rather than these differences being gender specific. Despite being mostly an older population, they did not feel that specific activities of daily living were men’s or women’s jobs but that these roles were shared, particularly after a couple retired.

Bowling states that there are four steps involved in answering questionnaires: comprehension of the question, recall of requested information from memory, evaluation of the link between the retrieved information and the question, and communication of the response [24]. The most burdensome modes are likely to be visual and written methods of self-administration, as these demand that respondents are literate in reading the language/s of the survey, that they do not have visual impairments and have the dexterity (e.g. of wrist and fingers) to complete the questions. The respondents’ reports of wanting further detail to answer the questions; for example, in wanting a directional component to ‘does it change your back pain?’ suggests consideration needs to be given to the relative benefits and disadvantages of self-report which have the advantage of being cost-effective and self-paced but may be subjection to unintended interpretation of questions. The alternative of a GP or health care professional administering the questions in person allows clarification of any ambiguity but is costly and time-consuming.

The research highlighted that some respondents disliked the binary nature of questions such as being asked to choose yes or no to whether they would describe their pain as sharp [Q6], like toothache [Q7], or whether walking increased their pain. In total, 8 of the 15 questions in the Vfrac screening tool required a binary yes or no response. The use of dichotomous scales (binary responses) in which two opposing responses are provided (typically ‘Yes’ or ‘No’) is common in health surveys and was chosen as it was felt they were easy for the respondents to understand and easily adapted to an electronic format for simple scoring. They also place less demand on respondents’ time and have lower cost involved in analysing the data [25].

Our findings highlight that understanding and answering questions is a complex process embedded in meaning making: ‘asking and answering questions is at once a simple and a subtle affair’ [26]. Questions and answers are grounded in an understanding of culturally shared language, and yet even in the same language, answering questions always incorporates a level of interpretation. We cannot assume that a question means the same thing to all respondents. Good qualitative research incorporates the freedom to change the question to clarify meaning, and it shines light onto the discursive and ambiguous nature of dialogue. This issue is exacerbated across language and should be considered in the design and interpretation of diagnostic and screening surveys.

Consideration needs to be given to the evolving nature of primary care consultations. There has been a move away from purely face-to-face consultations with a GP to more remote consultations by videoconferencing or telephone (35% of all consultations in March 2024 were remote) [27]). With telephone consultations, it is more likely that patients will complete the screening tool independently prior to a remote consultation with the GP. Therefore, it is possible that the length of consultation time needs to be adjusted to accommodate the need for discussion of the responses to the screening tool, depending on whether the patient has already responded to the questions in advance or not.

We also gained some interesting insights into men’s experience and how they characterised their pain and physical symptoms from their VFF. It is notable that in women, the pain descriptors such as ‘toothache’, stabbing, and sharp have been found to have some predictive value [12]. The authors found that pain which reached a crescendo was associated with VFF, whereas pain that was described as like toothache, or sharp was less likely to be due to VFF [12]. In our interviews that pain could not be characterised into one of these ‘types’ and a more complex and dynamic pattern of presentation was described.

There are limitations to this study. The purposive sampling used in this study achieved a sample of men of varying age and varying levels of physical activity. It is recognised however that the study drew on a relatively small sample of White English-speaking participants. These participants had previously volunteered to participate in the earlier Vfrac study and subsequently also volunteered again to participate in the qualitative study.

It is known that there are differences between those able and willing to participate in medical research studies and those who are not [28]. The research also took place in one health care setting, the NHS in England. Further research is required to explore the factors identified in this study and to explore these factors in non-NHS settings and other countries.

## Conclusion

This research has allowed the male perspective of osteoporosis to be heard and importantly identified that the Vfrac tool had no gender-specific barriers when used in men. No gender-specific changes were felt necessary for it to be used by both men and women.

It highlighted the trade-offs that arise from decisions made to make a questionnaire less burdensome to complete by patients or in conjunction with health care professionals by using binary responses to questions with the increased need for some respondents to frame the question before answering and the resultant less nuanced response.

## Supplementary Information

Below is the link to the electronic supplementary material.

Supplementary file1 (DOCX 18.7 KB)

Supplementary file2 (DOCX 52.5 KB)
