# Supplementary material for: Applicability of Vfrac in men: a qualitative study of an osteoporotic vertebral fracture screening tool for use in older people with back pain
Source: Arch Osteoporos. 2024 Nov 19;19(1):117. doi: 10.1007/s11657-024-01470-8 (PMC11576809; doi:10.1007/s11657-024-01470-8)
Supplement: Supplementary file 2 — Supplementary file2 (DOCX 52.5 KB) [file 11657_2024_1470_MOESM2_ESM.docx]

**Applicability of Vfrac In Men: An Osteoporotic Vertebral Fracture**

**Topic guide**

**Aims and objectives**

To determine if VFRAC questionnaire developed to help GPs identify broken bones in the spine (vertebral fractures) can be used in men. Information will be used to make changes to the questionnaire if necessary.

**Topics covered will be:**

- Find out a bit about you
- Understand experiences of pain and other symptoms
- Understand views of the wording and questions in current Vfrac questionnaire

**Introduction**

- Introduce self
- Introduce the study: who is it for, what is it about
- Key points:
- Length of interview – Around 1 – 1.5 hours
- Voluntary nature of participation and right to withdraw
- Recording of the interview
- Confidentiality and how findings will be reported
- No names
- Report and academic publications
- Short quotes from them in write-up
- Any questions that they have
- Reaffirm that consent form signed

**Part 1: About you**

- Age
- Ethnicity
- How many vertebral fractures
- Bone health – osteoporosis, any other fractures
- Any other conditions
- What do on a daily basis

**Part 2: Experience of pain and other symptoms**

1. When did you first notice something was wrong?
2. What did you think caused it?
3. What did it feel like?
4. How would you describe pain?

- What words use to describe pain?

Probes:

- - Crushing
  - Tingling
  - Squeezing
- How strong is pain? Describe feelings.
- Where is pain?
- How long experienced pain?
- How has pain changed since you first broke a bone in your spine, if at all?

1. Anything that makes pain better?

Probes:

- - Lying down
  - Staying still

1. Anything that makes pain worse?

Probes:

- - Damp
  - Cold
  - Exercise

1. How does pain change depending on what you do, if at all?

Probes:

- - Physical activity
  - Busy day/ day off

1. Can you describe any other symptoms you have you experienced, if any?

Probes:

- - Breathlessness
  - Shorter
  - Reduced appetite
  - Fatigue

1. Have you experienced other bad pain in another part of body?
2. If yes, what are the differences between that pain and your back pain?
3. If yes, what happened to your back pain when you experienced this?

**Part 3: Views of wording and questions in current Vfrac questionnaire**

1. Ask the participant to read through the Vfrac questionnaire and talk you through what they are thinking.

Prompt for feedback if not covered:

- How easy questions are to understand
- How easy are questions to answer
- Improvements to wording
- How relevant activities are to you
- Any examples of activities you would prefer
- How easy diagrams are to understand
- Improvements to diagrams

1. What do you think works well about this questionnaire?
2. What do you think works less well about this questionnaire?
3. What improvements would you make?
4. Is there anything that would put you off using it if a GP/healthcare professional suggested it?

**Part 4: Close**

1. Are there any other things or insights that you would like to tell me about today?
2. Do you have any questions for me about the project?
3. Thank them
4. Reaffirm confidentiality
